# Improving the Performance of a Triboelectric Nanogenerator by Using an Asymmetric TiO_2_/PDMS Composite Layer

**DOI:** 10.3390/nano13050832

**Published:** 2023-02-23

**Authors:** Qingyang Zhou, Ryuto Takita, Takashi Ikuno

**Affiliations:** Graduate School of Advanced Engineering, Department of Applied Electronics, Tokyo University of Science, Katsushika, Tokyo 125-8585, Japan

**Keywords:** triboelectric nanogenerator, TiO_2_/PDMS composite film, asymmetrical geometry, high dielectric constant, interfacial recombination

## Abstract

To improve the output power of the polydimethylsiloxane (PDMS)-based triboelectric nanogenerators (TENGs), we fabricated an asymmetric TiO_2_/PDMS composite film in which a pure PDMS thin film was deposited as a capping layer on a TiO_2_ nanoparticles (NPs)-embedded PDMS composite film. Although in the absence of the capping layer, the output power decreased when the content of TiO_2_ NPs exceeded a certain value, the asymmetric TiO_2_/PDMS composite films showed that the output power increased with increasing content. The maximum output power density was approximately 0.28 W/m^2^ at a TiO_2_ content of 20 vol.%. The capping layer could be responsible not only for maintaining the high dielectric constant of the composite film but also for suppressing interfacial recombination. To further improve the output power, we applied a corona discharge treatment to the asymmetric film and measured the output power at a measurement frequency of 5 Hz. The maximum output power density was approximately 78 W/m^2^. The idea of the asymmetric geometry of the composite film should be applicable to various combinations of materials for TENGs.

## 1. Introduction

Triboelectric nanogenerators (TENGs) are micro-generators that can effectively convert low-frequency mechanical energy wasted in the environment into electrical energy [1,2]. Among vibration power generation devices, TENGs have simple structures and are mechanically flexible [3,4]. They are used to power wearable electronic devices such as light-emitting diodes [5,6], calculators [7,8,9], watches [10], and sensors [11]. Due to the high voltage of TENGs, they can also be used as portable high-voltage power supplies [2,12,13,14]. The electrical signals from TENGs can also be used as self-powered sensors for real-time monitoring of vital signs [15,16], human movements [10,17], and environmental conditions [18,19]. They have great potential for applications in human machine interfaces (HMIs), the Internet of Things (IoT), wearables, and implantable fields [20,21,22]. Due to their unique mechanical adaptability at low frequencies [23], TENGs also have significant advantages in the field of blue energy [15,24,25]. The collected mechanical energy can provide power for a variety of electrochemical processes [26] and form a self-sustaining electrochemical system [27].

To date, there are mainly three approaches to improving the output of TENGs: selection of hetero-materials [28,29], modification of surface morphologies [30,31,32,33], and embedding high dielectric constant materials into polymer films [30,34,35,36]. The embedding method can strengthen the dielectric polarization by embedding a dielectric with a high relative permittivity into polymers used as friction materials [37]. Polydimethylsiloxane (PDMS) is the most commonly used material for triboelectric power generation due to its high electronegativity, flexibility, transparency, and cost-effectiveness and can be easily formed into composite films by mixing nanoparticles (NPs) and other nanostructures [38,39]. This improvement is due to new materials developed by surface patterning or functional group attachment [27].

There are reports of embedding TiO_2_ NPs, BaTiO_3_ NPs, and SrTiO_3_ NPs, which are high-*k* materials, to improve output [27,35,40] because the relative permittivities of composite films determine the generated charge densities, which are proportional to the output power [41]. Park et al. achieved an output voltage of 180 V and an output power density of 1.84 W/m^2^ by embedding the polycrystalline rutile TiO_2_ NPs. The output voltage was 1.6 times higher than that from the film without TiO_2_ [27]. Chen et al. achieved an output voltage of 338 V and an output power of 6.47 W/m^2^ by embedding the SrTiO_3_ NPs and micropores. The output voltage was twice as high as without this embedding [41]. What they all have in common is that, while the output power increased as the content of high-*k* materials in the PDMS composite film increased, the output suddenly decreased when the content exceeded a certain value. The cause of this problem is not yet fully understood. Moreover, no report has yet been published to solve this problem.

To clarify the reason for the drop in output due to the excessive amount of embedding, we characterized the surface structure using a scanning electron microscope (SEM). Based on the results, we fabricated an asymmetric TiO_2_/PDMS composite film to solve the problem. The result was that the output increased with increasing amounts of TiO_2_ NPs. The maximum power density was about twice that of the uncoated sample. To further improve the output, we optimized the measurement conditions and applied a corona discharge treatment [42] to the asymmetric film, resulting in a maximum output density of approximately 78 W/m^2^.

## 2. Materials and Methods

We synthesized PDMS-based composite films with a square of 50 mm and a thickness of 0.5 mm, in which anatase TiO_2_ NPs (FUJIFILM Wako Pure Chemicals Corporation, Ltd., CAS 1317-70-0, Osaka, Japan) with an average diameter of approximately 100 nm were embedded. The reason why we chose TiO_2_ particles from among many high-*k* materials is that TiO_2_ is an inexpensive binary oxide semiconductor with well-known physical properties. First, the source materials of the PDMS films were the base and agent of the SILPOT 184 (Dow Chemicals), and the TiO_2_ NPs were mixed with a magnetic stirrer (AS ONE Corporation, Ltd., REXIM RS-4D, Osaka, Japan) at a rotation speed of 800–1200 rpm for 5 min at room temperature (RT). The contents of TiO_2_ NPs ranged from 0.5 to 20 vol.%. Then, the air bubbles in the mixture were removed using a rotary pump (AS ONE Corporation, Ltd., AOP42C, Osaka, Japan). The mixed solution was then poured into an aluminum (Al) mold, which has a square groove of 55 mm per side and a depth of 0.5 mm. The mold containing the solution was heated at 100 °C for 1 h in an oven (AS ONE Corporation Ltd., HTO-450S, Osaka, Japan) under atmospheric pressure. After cooling to RT, the composite film was peeled off from the Al mold. When the TiO_2_ content in PDMS exceeded 20%, the film became mechanically fragile, and its power generation properties could not be measured.

**Figure 1:**
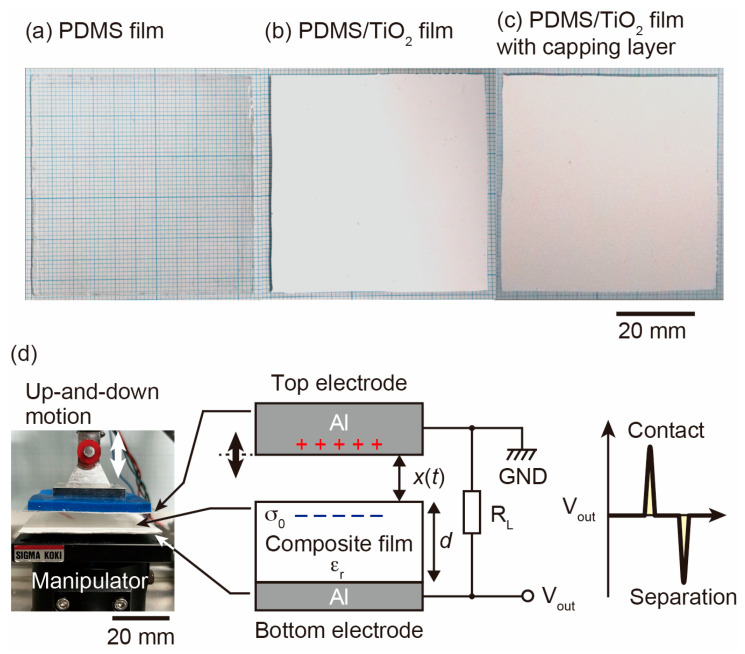
Photographs of (**a**) PDMS film, (**b**) PDMS/TiO_2_ film, and (**c**) PDMS/TiO_2_ film with a PDMS capping layer. (**d**) A schematic of the apparatus used to characterize the power generation and the typical profile of the output voltage.

Figure 1a,b show photographs of the composite films without and with the TiO_2_ NPs, respectively. The composite film without the TiO_2_ NPs was transparent to the naked eye, while the composite film with the TiO_2_ NPs had an opaque white color. We then applied a pure PDMS film as a capping layer on the composite film to investigate the effects of the capping layer on the output power. The capping layer was prepared using a spin coater (MIKASA, MS-B100, Tokyo, Japan). The spin coating time and rotation speed were 60 s and 1250 rpm, respectively. The thickness of the capping layer was approximately 0.05 mm. Figure 1c shows a photograph of the composite film with the PDMS capping layer. Comparing the composite film without the capping layer with the one with the capping layer, they are almost identical to the naked eye.

To increase the output power, negative charges were injected into the composite films using a corona discharge gun (Green Techno, GC90N, Kanagawa, Japan). The distance between the charging gun and the sample was approximately 4 cm. The discharge time was 60 s. The heating temperature was 180 °C. The discharge voltage during charge injection was −35 kV. The details of the injection experiment were described elsewhere [42].

After fabrication of the composite films, we characterized the relative permittivity. Circular gold (Au) thin films with a diameter of 1.5 mm and a thickness of approximately 30 nm were deposited on the composite films using a thermal evaporator (ULVAC SINKU KIKO, VPC-410, Kanagawa, Japan) as the measuring electrodes. We prepared nine samples for each type of TiO_2_ NP. The relative permittivities of the films were measured using an LCR meter (HIOKI, IM3536, Nagano, Japan) and a probe (APOLLOWAVE, KB20200100, Osaka, Japan). To investigate the presence of TiO_2_ NPs on the surface of the composite film, we used a SEM (Carl Zeiss, SUPRA40, Jena, Germany) at different acceleration voltages of 3.5 and 9 kV. Before SEM observations, we applied an osmium plasma coating to avoid the charging effect using an osmium coater (Meiwafosis Co., Ltd., Neoc-Pro, Tokyo, Japan).

Figure 1d shows a photograph and a schematic of the apparatus used to characterize the power generation and a typical profile of the output voltage when the composite film and the Al counter electrode are in contact and separated. When the top electrode and the composite film are in contact, positive and negative charges are induced on the top Al counter electrode and the surface of the composite film, respectively. The current flows through the load resistance *R*_L_, and a positive voltage is generated. Then, when the top Al counter electrode and the composite film are separated, the current flows in the opposite direction, and a negative voltage is generated [42]. We used a resistor of 1 MΩ as the *R*_L_. An oscilloscope (GWINSTEK, GDS-1074B, Taipei, Taiwan) was used to measure the voltage across the *R*_L_. We changed the frequencies of the mechanical contact to between 2 and 5 Hz. All measurements were carried out at RT and in air.

## 3. Results and Discussion

**Figure 2:**
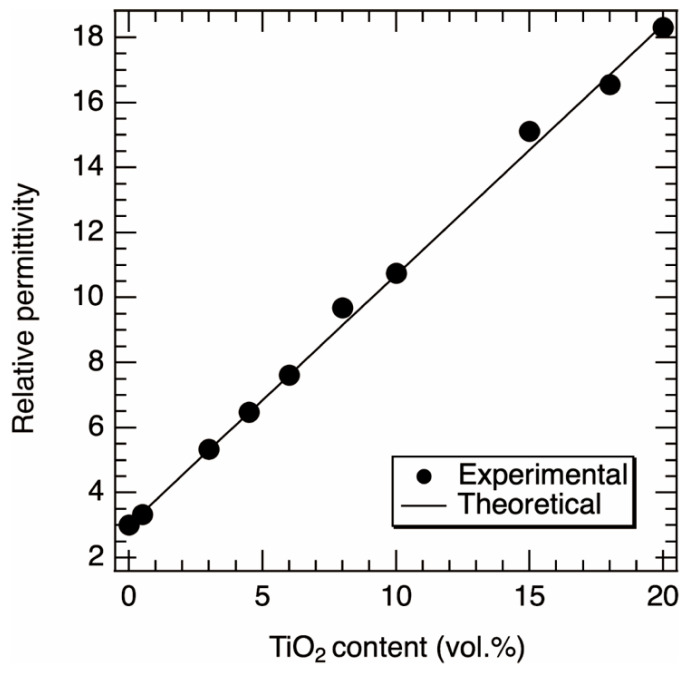
Comparison of theoretical and experimental values of relative permittivity of composite films.

Theoretically, it is known that the output voltage increases with increasing the relative permittivity *ε*_r_ or decreasing the thickness of the composite film, or both [41]. The open circuit voltage, *V*_oc_, is given by *V*_oc_ = *σ*_0_·*x*(*t*)/*ε*_0_, where *σ*_0_, *x*, *t*, and *ε*_0_ are the triboric charge density on the composite film, the gap between the top electrode and the composite film, time, and the vacuum permittivity, respectively. The *σ*_0_ depends on the capacitance of the device for contact-mode tribo-generators. Namely, increasing the *ε*_r_ is one of the routes to increasing the output voltage because the maximum capacitance, *C*_max_, of the device is described by *C*_max_ = *ε*_0_·*ε*_r_·*S*/*d*, where *S* is the surface area of the composite film [41]. We characterized the relative permittivity *ε*_r_ of PDMS/TiO_2_ composite films without a capping layer as a function of the content of TiO_2_ NPs. Figure 2 shows the relationship between the experimental values of *ε*_r_ as a function of the content of TiO_2_ NPs. The *ε*_r_ was almost linearly proportional to the content of TiO_2_ NPs. The pure PDMS showed a *ε*_r_ of 3.02, indicating that this value is almost in agreement with the literature value of 3 [41]. When the content of the TiO_2_ NPs was 20 vol.%, the *ε*_r_ was 18.31. The mean square errors of the average values were found to be less than 3% of the average value.

The theoretical values of *ε*_r_ for the composite film can be estimated using the following equation

(1) $$\varepsilon_{r}=\varepsilon_{r\left({\mathrm{TiO}}_{2}\right)}f_{\left({\mathrm{TiO}}_{2}\right)}+\varepsilon_{r\left(\mathrm{PDMS}\right)}f_{\left(\mathrm{PDMS}\right)}$$

where *ε*_r(TiO_2_)_ and *ε*_r(PDMS)_ are the literature values of the relative permittivity of TiO_2_ and PDMS, respectively (*ε*_r(TiO_2_)_ = 80 and *ε*_r(PDMS)_ = 3) [41], and *f*_(TiO_2_)_ and *f*_(PDMS)_ are the contents of TiO_2_ NPs and PDMS, respectively. The theoretical relative permittivity as a function of TiO_2_ content is also shown in Figure 2. The slope of the line was approximately 0.77. It was found that the theoretical line was in agreement with the experimental values. Therefore, we confirmed that the *ε*_r_ of the composite film can be accurately controlled by our fabrication method.

**Figure 3:**
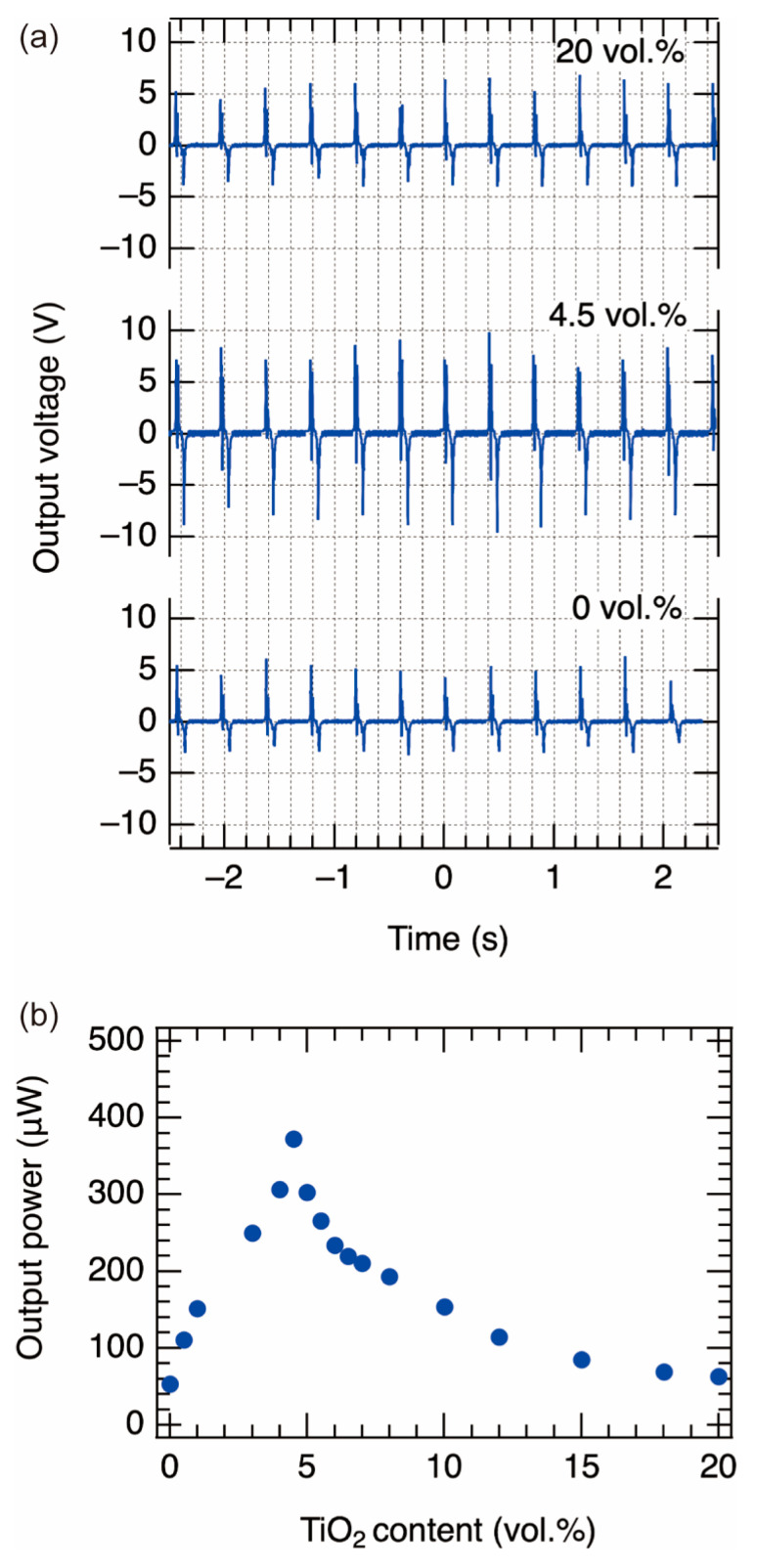
(**a**) The power generation waveform of the composite film shown on the oscilloscope. (**b**) The relationship between the content of TiO_2_ NPs and the output power.

Next, we measured the triboelectric power generation of the composite films. The measured films had TiO_2_ NP contents ranging from 0 to 20 vol.%. Figure 3a shows the voltage waveforms of the samples with different contents of TiO_2_. The repetitive voltage peaks were observed stably. It seems that the peak-to-peak voltage of the sample with a content of 4.5 vol.% was larger than that of the samples with 0 and 20 vol.%.

To investigate the relationship between TiO_2_ content and output power, we estimated the output powers using the average value of 10,000 peak-to-peak voltages. Figure 3b shows the relationship between the content of TiO_2_ NPs and the output power. The output power increased with increasing content of TiO_2_ NPs in the range of 0 to 4.5 vol.%. The output power of the pure PDMS was 53.29 μW, whereas the maximum output power was 372.49 μW (a power density of 0.15 W/m^2^) for the sample containing 4.5 vol.%, which is seven times higher than that of the pure PDMS. In contrast, the output power was found to decrease when the content exceeded 4.5%. Although we expected that as the TiO_2_ content increased, the relative permittivity of the composite film would increase, resulting in an increase in output power, in fact, the output power peaked at around 4.5 vol.%. In a previous study [41], a similar trend was found, and it was argued that the output power decreases with increasing TiO_2_ content because the effective friction area of PDMS is smaller when the TiO_2_ content is higher. We further investigated the cause of the decreasing trend. We assumed charge recombination at the TiO_2_/Al interface due to the surface exposure of TiO_2_ NPs. As discussed later, there was no decrease in the output power when a pure PDMS film was coated on the TiO_2_/PDMS composite film. Therefore, the TiO_2_/PDMS composite film surface is the cause of the reduction in output.

**Figure 4:**
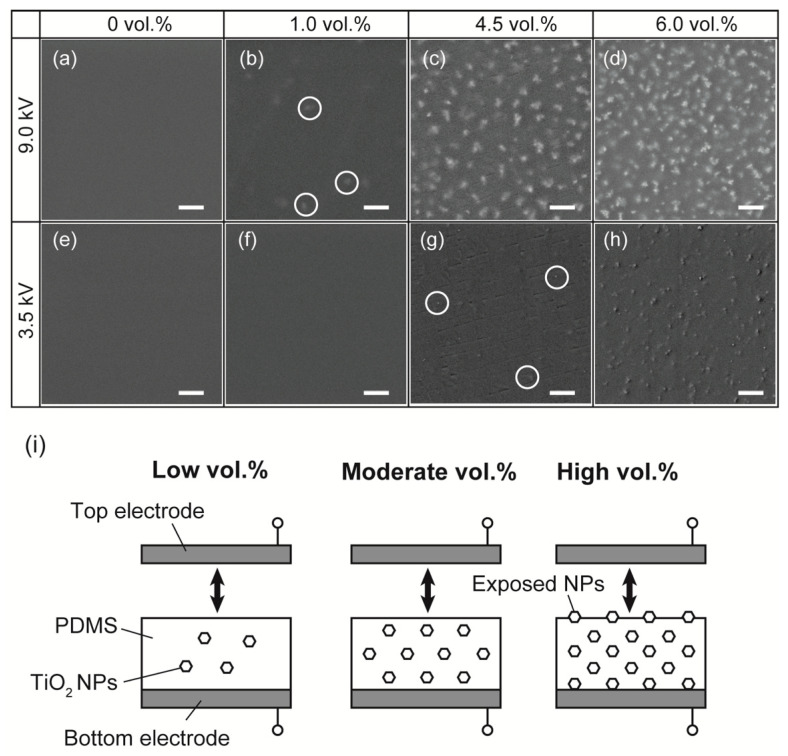
(**a**–**d**) SEM images of each content of TiO_2_ NPs at acceleration voltages of 9 kV and (**e**–**h**) at acceleration voltages of 3.5 kV. The scale bars of the SEM images are 1 μm. (**i**) The structure of the composite films with the different contents of TiO_2_ NPs.

We observed the surface of the composite films to confirm the exposure of the TiO_2_ NPs. The acceleration voltages of 3.5 and 9.0 kV were chosen for the SEM observation to obtain micro-structural information at different depths. The acceleration voltage of 3.5 kV was more sensitive to the surface of the films than that of 9.0 kV. Figure S1 in the supporting information shows electron trajectories in the PDMS/TiO_2_ composite. From the simulation, for an acceleration voltage of 3.5 kV, the maximum number of collisions was approximately 80 nm. This indicates that the SEM image shows information in the vicinity of the surface. For the acceleration voltage of 9.0 kV, the number of collisions was almost the same within the depth of 1 μm. In other words, the SEM images show information not only on the surface but also in deeper regions. Figure 4a–d show the SEM images of the composite films with different TiO_2_ contents at the accelerating voltage of 9.0 kV. Almost no white dots were observed up to a TiO_2_ NP content of 1 vol.%, while white dots were observed at a TiO_2_ NP content of more than 1 vol.%. In contrast, almost no structures with white dots were observed at an accelerating voltage of 3.5 kV, up to a content of TiO_2_ NPs of 4.5 vol.% as shown in Figure 4e,f. On the contrary, at a content of TiO_2_-NP of 4.5 vol.%, white dots were observed, which are indicated in the circles of Figure 4g. At a content higher than 4.5 vol.%, many white dot structures were observed, as shown in Figure 4h. This indicates that the density of the TiO_2_ NPs exposed on the surface increased when the content of TiO_2_ NPs exceeded 4.5 vol.%.

Based on the SEM observation, we can speculate on the structure of the composite films. Films with a low content of TiO_2_ NPs, such as those in Figure 4b,f, could have the cross-sectional structure shown on the left side in Figure 4i. The structure of films with moderate content of TiO_2_ NPs, such as those in Figure 4c,g, was shown in the middle of Figure 4i, and the structure of composite films with a high content of TiO_2_ NPs, such as those in Figure 4d,h, was shown on the right side of Figure 4i. As shown in Figure 4i, when the content of TiO_2_ NPs was low or moderate, the TiO_2_ NPs were inside the composite films, but when the content of TiO_2_ NPs was high, the TiO_2_ NPs were exposed on the surface of the composite films.

**Figure 5:**
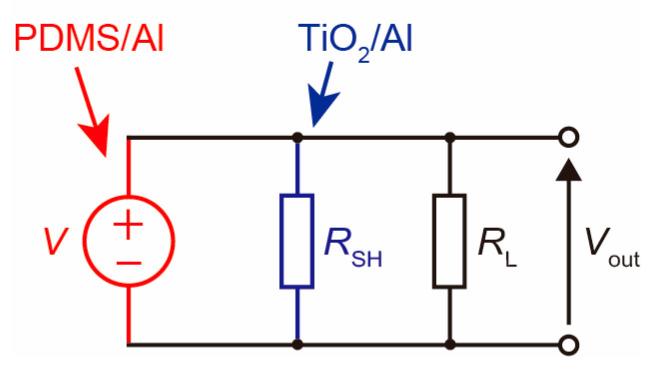
The equivalent circuit during triboelectric power generation.

When there is excess TiO_2_ in the PDMS film, it was found that the TiO_2_ NPs are exposed on the surface of the composite film. This means that when the composite film comes into contact with the Al top electrode during power generation, the Al should come into contact not only with the PDMS but also with the TiO_2_ NPs. To understand the carrier dynamics, we calculated the band diagram of the TiO_2_/Al interface using a semiconductor simulator named the Solar Cell Capacitance Simulator (SCAPS 3.3.10) (see Figure S1 in the Supporting Information). Since the TiO_2_ used in this study was made of nanoparticles, it is difficult to know the exact physical properties. Therefore, the following bulk properties were used for the simulation: the band gap is 3.1 eV [43], the electron affinity is 4.0 eV [44], the relative permittivity is 80 [41], the conduction band effective density of states is 2.2 × 10^18^ [45], the valence band effective density of states is 1.8 × 10^19^ [45], and the shallow uniform donor density is 10^17^. We used the electron affinity of Al of 4.06 eV [46]. As a result of the simulation, we found that the band of TiO_2_ bent downward with a diffusion potential of 0.02 eV. This means that the TiO_2_/Al interface plays the role of an ohmic junction. Although polarization occurs at the interface between PDMS and Al, which is the commonly proposed power generation mechanism for triboelectric devices [1], the charges generated at the PDMS/Al interface might recombine at the TiO_2_/Al interface, resulting in a reduction in the number of charges that determine the magnitude of output. Figure 5 shows the equivalent circuit from the speculation mentioned above, where the PDMS/Al interface is the voltage source *V* and the TiO_2_/Al interface is the shunt resistance *R*_SH_. The output voltage *V*_out_ is the voltage across the load resistor *R*_L_. The decrease in output when the TiO_2_ content exceeds 4.5%, as shown in Figure 3b, can be attributed to an increase in the amount of exposed TiO_2_ and consequently a decrease in *R*_SH_.

**Figure 6:**
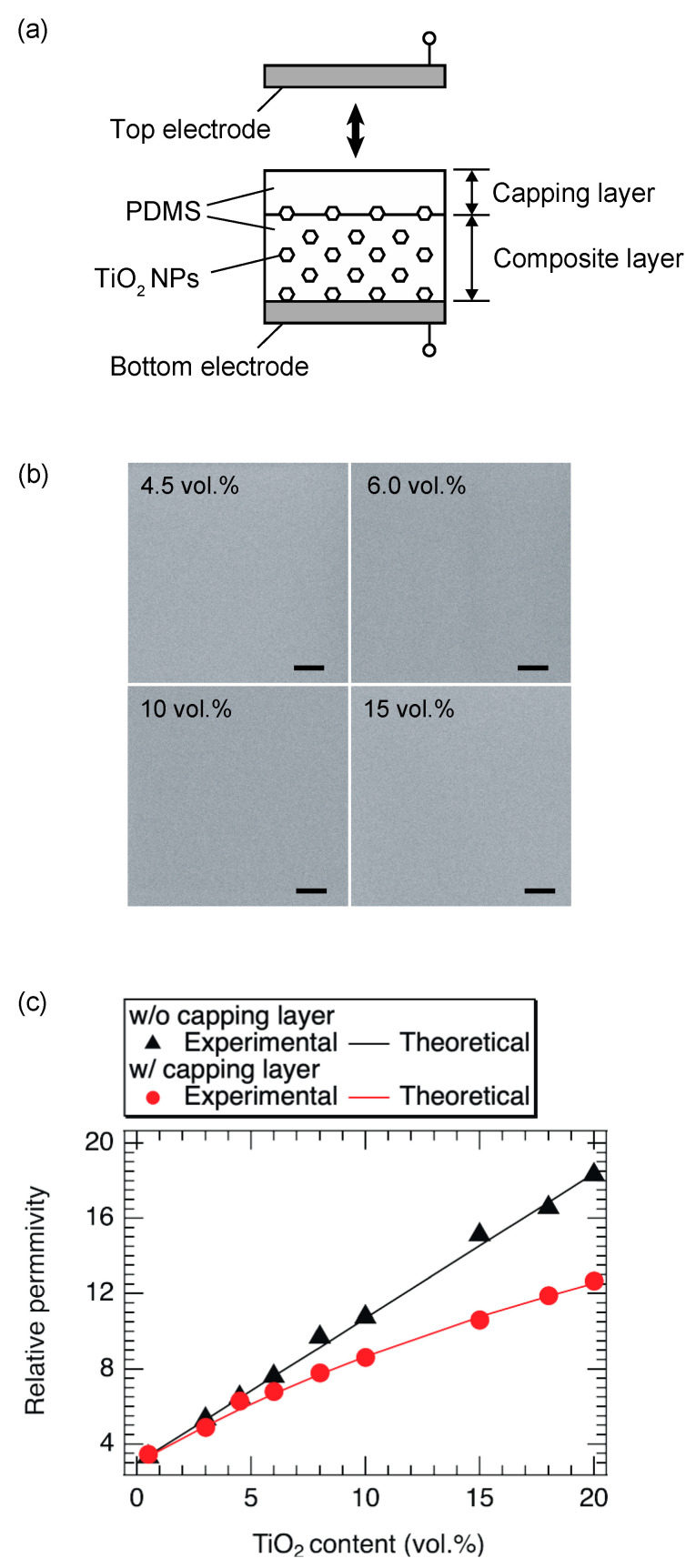
(**a**) The structure of the film after spin coating. (**b**) SEM images of the contents of the TiO_2_ NPs at an accelerating voltage of 3.5 kV. The scale bars of the SEM images are 1 μm. (**c**) The relationship between the relative permittivity and the content of TiO_2_ NPs in the films with and without the capping layer.

To prevent recombination, we coated the surface of the composite film with a PDMS thin film, called the capping layer, as shown in Figure 6a. We call the ensemble an asymmetric PDMS/TiO_2_ film. The thickness of the capping layer was approximately 0.05 mm. We observed the surface structure of the asymmetric films using SEM at an acceleration voltage of 3.5 kV. Figure 6b shows the top-view SEM images of the asymmetric composite films with the capping layer for different TiO_2_ contents. No white dot structures were observed in these samples, although the samples without a capping layer in Figure 4g,h had white dots. Therefore, we confirmed that no TiO_2_ NPs could be exposed on the surface of the asymmetric composite film by coating the capping layer. Next, the relative permittivity of the films was measured. Figure 6c shows the relative permittivity of the films with and without the capping layers as a function of TiO_2_ content. The mean square errors of the average values were found to be less than 3% of the average value. Even for composite films with the same TiO_2_ content, the relative permittivity of the film with the capping layer was lower than that of the film without the capping layer. The relationship between the relative permittivity and TiO_2_ content was linear in the absence of the capping layer but nonlinear in the presence of the capping layer.

The theoretical values of *ε*_r_ of the composite film with a capping layer can be estimated from the following equation

(2) $$\varepsilon_{r}=\frac{\varepsilon_{\left(\mathrm{PDMS}\right)}\cdot \varepsilon_{\left(\mathrm{PDMS}+{\mathrm{TiO}}_{2}\right)}\cdot \left\{d_{\left(\mathrm{PDMS}\right)}+d_{\left(\mathrm{PDMS}+{\mathrm{TiO}}_{2}\right)}\right\}}{\varepsilon_{\left(\mathrm{PDMS}\right)}\cdot d_{\left(\mathrm{PDMS}+{\mathrm{TiO}}_{2}\right)}+\varepsilon_{\left(\mathrm{PDMS}+{\mathrm{TiO}}_{2}\right)}\cdot d_{\left(\mathrm{PDMS}\right)}}$$

where *d*_(PDMS)_ and *d*_(PDMS+TiO_2_)_ are the film thicknesses of the capping layer and the composite layer, respectively, and *ε*_(PDMS)_ and *ε*_(PDMS+TiO_2_)_ are the relative permittivity of the capping layer and the composite layer, respectively. The theoretical curve is represented by the solid red line in Figure 6c. It was found that the curve agreed well with the experimental values.

**Figure 7:**
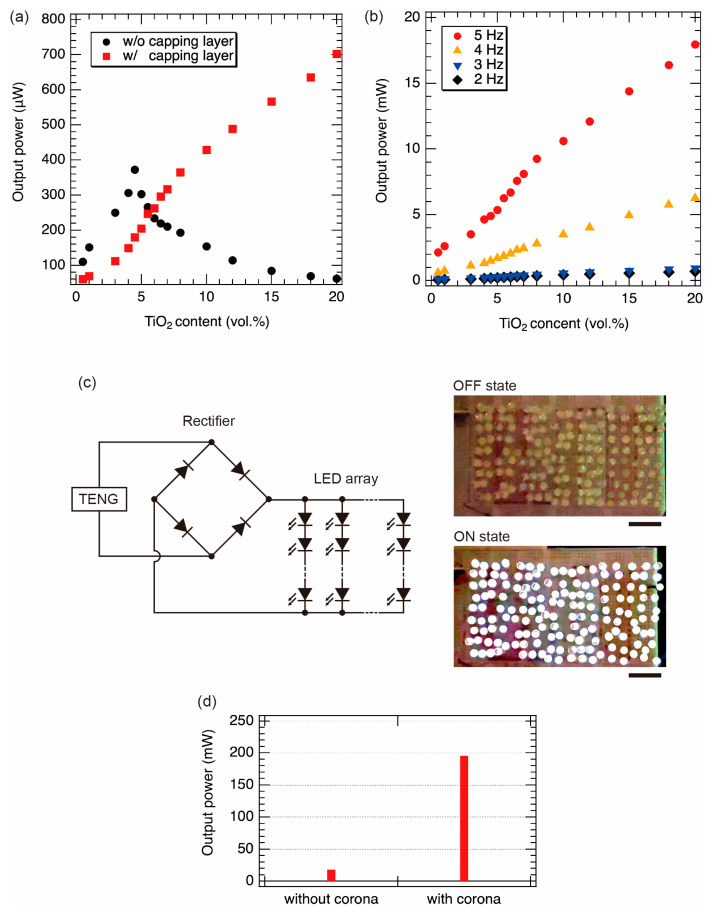
(**a**) The relationship between the content of TiO_2_ NPs and the output power. (**b**) The output power at the frequencies of 2, 3, 4, and 5 Hz. (**c**) The schematic circuit of TENG connections with an LED array. Photographs of LED array for OFF and ON states. Scale bars are 2 cm. (**d**) The comparison between the output power with and without corona discharge.

Next, we measured the output powers of the films with the capping layers as a function of the content of TiO_2_ NPs. Figure 7a shows the output power as a function of TiO_2_ content. Although the output power decreased when the content of TiO_2_ NPs exceeded 4.5 vol.% for the composite films without the capping layers, the output power was successfully increased with the content of TiO_2_ NPs by coating the capping layer. When the content of TiO_2_ NPs exceeded 10 vol.%, the output power was higher than that of the sample without the capping layer. The maximum output power was approximately 702.25 μW (a power density of 0.28 W/m^2^) at the content of the TiO_2_ NPs of 20 vol.%. The reason for the low output power at a content of less than 10 vol.% is probably due to the low relative permittivity of the composite film. Although the relative permittivity was lowered by coating the capping layer, as shown in Figure 6c, the surface recombination was reduced, as shown in Figure 5, resulting in an increase in output power.

We also investigated the effect of contact/separation frequency on the output power in triboelectric power generation. Figure 7b shows the output power as a function of the TiO_2_ content at different frequencies. The maximum output power was approximately 18 mW (a power density of 7.2 W/m^2^) at a frequency of 5 Hz. The sample with the highest output (a PDMS capping layer on PDMS/TiO_2_ composite film (20 wt.%)) was used to demonstrate LED luminescence. Figure 7c shows a schematic circuit of this demonstration. TENG in Figure 7c means the asymmetric composite film. TENG was connected to the bridge diode. The output was rectified by a bridge diode to power an array of 156 LEDs (12 series connections in 13 sets). The contact/separation frequency was 5 Hz. We confirmed that the LED array was found to emit light clearly and synchronously with the cycles of contact and separation.

Furthermore, to increase the output power, a corona discharge treatment was also performed. We have already reported that the corona discharge treatment for the co-tribo layer is effective in boosting the output power. Negative charges can be fixed to the composite film by corona discharge treatment. This technique is often used in the fabrication of electrets. In addition to the polarization caused by triboelectric generation, the existence of fixed negative charges in the composite film has a superimposed effect of electrostatic induction on the counter electrode, an Al electrode. As a result, the power generation capability is greatly enhanced. Details are shown in our previous report [42]. Figure 7d shows the output powers with and without corona discharge at a measurement frequency of 5 Hz. The output power with the corona discharge was approximately 10 times higher than the output power without the corona discharge. The maximum output power was approximately 195 mW (a power density of 78 W/m^2^). Compared with previous studies [27,41], which also used TiO_2_ NPs and PDMS to generate triboelectricity, our output power was about four times higher than theirs.

## 4. Conclusions

In summary, to improve the output power of the PDMS-based TENGs, we fabricated asymmetric TiO_2_/PDMS composite films, in which a TiO_2_ NPs embedded PDMS film and a pure PDMS film as a capping layer are stacked. In the case of the composite film without a capping layer, although the output power increased with increasing content of TiO_2_ NPs in the range of 0 to 4.5 vol.%, the output power decreased when the content exceeded 4.5 vol.%. This trend is similar to the results reported previously [27,41]. We revealed that the decrease in the output power might be caused by the exposure of TiO_2_ NPs on the TiO_2_/PDMS composite film. To prevent the decrease in output power, we fabricated the asymmetric TiO_2_/PDMS composite films (the composite film with a capping layer). As a result, the output power was increased with increasing content of TiO_2_ NPs, and no decrease in the output power was observed. The maximum output power was approximately 702.25 μW (a power density of 0.28 W/m^2^) when the content of TiO_2_ NPs was 20 vol.%. The capping layer could be responsible not only for maintaining the high dielectric constant of the composite film but also suppressing interfacial recombination.

To further improve the output power, we treated the asymmetric film with a corona discharge and measured the output at a measurement frequency of 5 Hz. The maximum output power was approximately 195 mW (78 W/m^2^). We found that the surface capping layer effectively suppresses surface recombination when inorganic particle-embedded films are used as components of TENG to improve the relative permittivity. This method is expected to be applicable to various material combinations for TENGs.

## Data Availability

Not applicable.

## Supplementary Materials

The following supporting information can be downloaded at: https://www.mdpi.com/article/10.3390/nano13050832/s1, Figure S1: Monte Carlo simulation of electron trajectories in the TiO_2_/PDMS composite films. Penetration simulation in a material of 500 electrons entering from the position indicated by the red arrow with the acceleration voltage of (a) 3.5 kV and (b) 9.0 eV. (c) Number of collision vs. depth of the composite film, Figure S2: Band diagram around the interface of TiO_2_ and Al.
