# Supplementary material for: Improving the Performance of a Triboelectric Nanogenerator by Using an Asymmetric TiO2/PDMS Composite Layer
Source: Nanomaterials (Basel). 2023 Feb 23;13(5):832. doi: 10.3390/nano13050832 (PMC10005343; doi:10.3390/nano13050832)
Supplement: Supplementary file 1 [file nanomaterials-13-00832-s001.zip › nanomaterials-2206184-supplementary.pdf]

nanomaterials

Supplementary Materials

Improving the Performance of a Triboelectric Nanogenerator by Using an Asymmetric TiO_2/PDMS Composite Layer

Qingyang Zhou, Ryuto Takita and Takashi Ikuno *

Graduate School of Advanced Engineering, Department of Applied Electronics, Tokyo University of Science, Katsushika, Tokyo 125-8585, Japan
* Correspondence: tikuno@rs.tus.ac.jp

1. Penetration depth of incident electron beam with different acceleration voltages

Scanning electron microscope (SEM) observations were carried out using electron beams of different acceleration voltages to confirm the presence of TiO_2 nanoparticles (NPs) near the surface. The electron beam that penetrates into the material interacts with the material, and secondary electrons are emitted from the material by collision. This means that we can obtain information on the microstructure around the penetration depth of the electron beam.

Electron trajectories in the material were simulated by Monte-Carlo simulator developed by us. Figure S1a,b show electron trajectories in the TiO_2/PDMS composite films, of which acceleration voltages are 3.5 and 9.0 eV, respectively. Obviously, penetration depth of electron beam with 3.5 kV is lower than that with 9.0 kV. Figure S1c shows the number of collisions between electrons and materials as a function of the depth. For 3.5 kV, the maximum number of collisions was approximately 80 nm. This indicates that information in the vicinity of the surface can be observed. When the acceleration voltage was 9.0 kV, the number of collisions was almost the same within the entire region. This indicates that information is observed not only on the surface but also in deep regions.

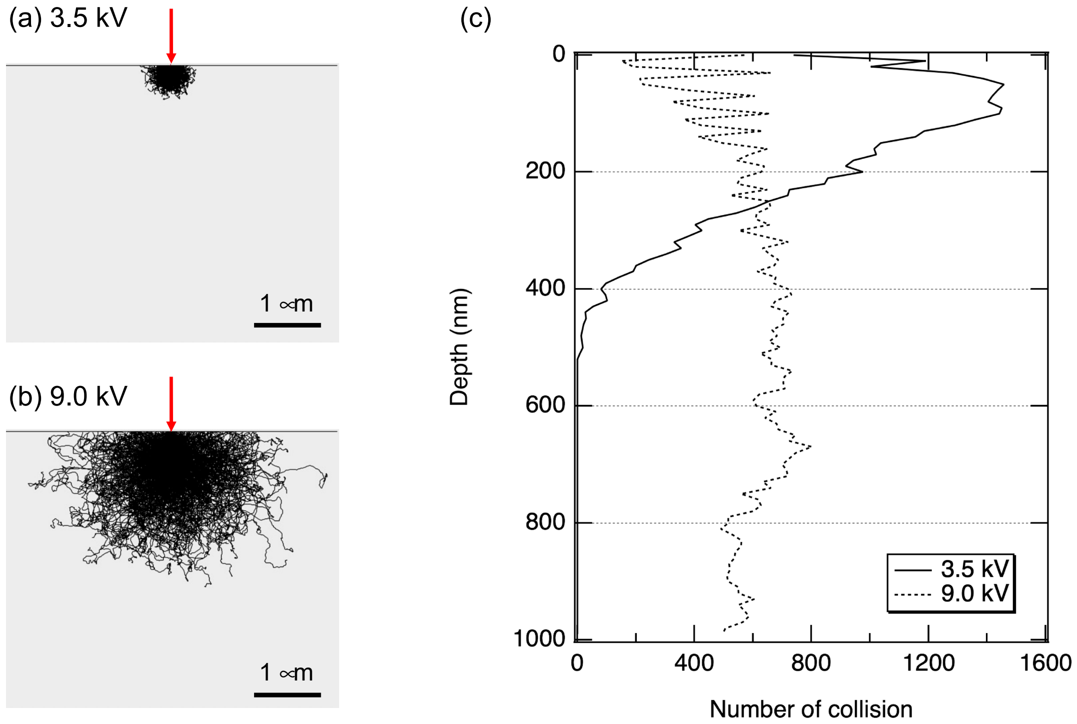

Figure S1. Monte Carlo simulation of electron trajectories in the TiO_2/PDMS composite films. Penetration simulation in a material of 500 electrons entering from the position indicated by the red

arrow with the acceleration voltage of (**a**) 3.5 kV and (**b**) 9.0 eV. (**c**) Number of collision vs. depth of the composite film.

2. Band bending at the interface of Al and TiO_2

We calculated the band diagram of Al/TiO_2 junction using a solar cell capacitances simulator (SCAPS 3.3.10). Material parameters of TiO_2 shown as follows below were used for the calculation: the band gap of 3.1 eV [1], the electron affinity of 4.0 eV [2], the relative permittivity of 80, a conduction band effective density of states of 2.2×10^{18} [3], a valence band effective density of states of 1.8×10^{19}[3], the electron thermal velocity of 10^7, the hole thermal velocity of 10^7, the electron mobility of 20 [3], the hole mobility of 10 [3], the shallow uniform donor density of 10^{17}, and the shallow uniform acceptor density of 0. The electron affinity of Al of 4.06 eV [4] was used. Figure S2 shows the band diagram around the interface of TiO_2 and Al. At the interface, the conduction band minimum (CBM) and valence band maximum (VBM) were bent because of the difference of work functions of TiO_2 and Al. The quantity of the bending was approximately 0.02 eV.

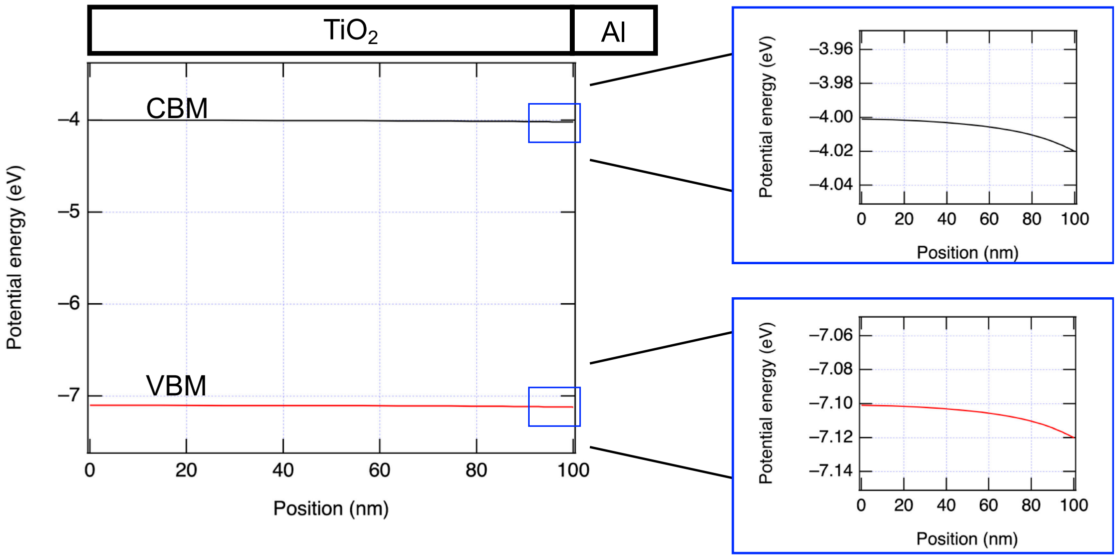

Figure S2. Band diagram around the interface of TiO_2 and Al.

References

1. Kim, H.S.; Lee, C.R.; Im, J.H.; Lee, K.B.; Moehl, T.; Marchioro, A.; Moon, S.J.; Humphry-Baker, R.; Yum, J.H.; Moser, J.E.; et al. Lead Iodide Perovskite Sensitized All-Solid-State Submicron Thin Film Mesoscopic Solar Cell with Efficiency Exceeding 9%. *Sci. Rep.* **2012**, *2*, 591. https://doi.org/10.1038/srep00591.
2. Etgar, L.; Gao, P.; Xue, Z.; Peng, Q.; Chandiran, A.K.; Liu, B.; Nazeeruddin, M.K.; Grätzel, M. Mesoscopic $CH_3NH_3PbI_3/TiO_2$ heterojunction solar cells. *J. Am. Chem. Soc.* **2012**, *134*, 17396–17399. https://doi.org/10.1021/ja307789s.
3. Jeyakumar, R.; Bag, A.; Nekovei, R.; Radhakrishnan, R. Influence of Electron Transport Layer (TiO2) Thickness and Its Doping Density on the Performance of CH3NH3PbI3-Based Planar Perovskite Solar Cells. J. Electron. Mater. 2020, 49, 3533–3539. https://doi.org/10.1007/s11664-020-08041-w.
4. Michaelson, H.B. The work function of the elements and its periodicity. J. Appl. Phys. 1977, 48, 4729. https://doi.org/10.1063/1.323539.
